# Bladder masses simulating neoplasia in chronic granulomatous disease: Diagnostic challenges and spontaneous regression

**DOI:** 10.1016/j.eucr.2025.103312

**Published:** 2025-12-08

**Authors:** Mehdi Dadpour, Niloofar Soleimanifard, Sobhan Alishah, Miaad Chalaki

**Affiliations:** a Department of Urology, Shahid Labbafinejad Medical Center, The Center of Excellence in Urology, Urology and Nephrology Research Center, Research Institute for Urology and Nephrology, Shahid Beheshti University of Medical Sciences, Tehran, Iran; b Department of Urology, Hasheminejad Kidney Center, School of Medicine, Iran University of Medical Sciences, Tehran, Iran

**Keywords:** Chronic granulomatous disease, Bladder neoplasm, Hematuria, Biopsy

## Abstract

Chronic granulomatous disease (CGD) rarely involves the lower urinary tract, and bladder mass–like lesions are exceptionally uncommon. We report a 23-year-old male with CGD who presented with gross hematuria and multiple large papillary bladder masses on imaging and cystoscopy. Cold-cup biopsies suggested papillary urothelial proliferation but were inconclusive due to limited sampling. Remarkably, repeat cystoscopy and MRI one month later showed complete resolution of all lesions. This case highlights the potential for CGD-related reactive or inflammatory changes to mimic neoplasia and emphasizes the importance of cautious interpretation and short-interval reassessment in such patients.

## 1 Introduction

Chronic granulomatous disease (CGD) is a rare primary immunodeficiency marked by impaired phagocytic function, predisposing patients to recurrent infections and granuloma formation across multiple organ systems.^1^ While gastrointestinal and respiratory involvement are well documented, urological manifestations are uncommon and usually arise from obstructive processes caused by granulomatous inflammation.^2^ The occurrence of bladder masses in individuals with CGD is exceptionally rare, and lesions with a neoplastic appearance present notable diagnostic challenge, particularly in young adults.^3^

Papillary urothelial tumors, including papillary urothelial neoplasm of low malignant potential (PUNLMP), are seldom seen in this age group, making their presence in an immunocompromised patient even more unexpected. Moreover, distinguishing true neoplastic changes from reactive or inflammatory mimics in CGD is often difficult, especially when biopsy specimens are limited.^4^^,^^5^

We describe a rare case of a young male with CGD who developed multiple bladder masses resembling urothelial neoplasia, which completely regressed within one month, underscoring important diagnostic and clinical considerations.

## 2 Case presentation

A 23-year-old male with a known history of chronic granulomatous disease (CGD) since childhood—initially presenting with granulomatous lesions of the buccal mucosa and axillary region—had been maintained on itraconazole, cotrimoxazole prophylaxis, and interferon-gamma injections.

**Fig. 1:**
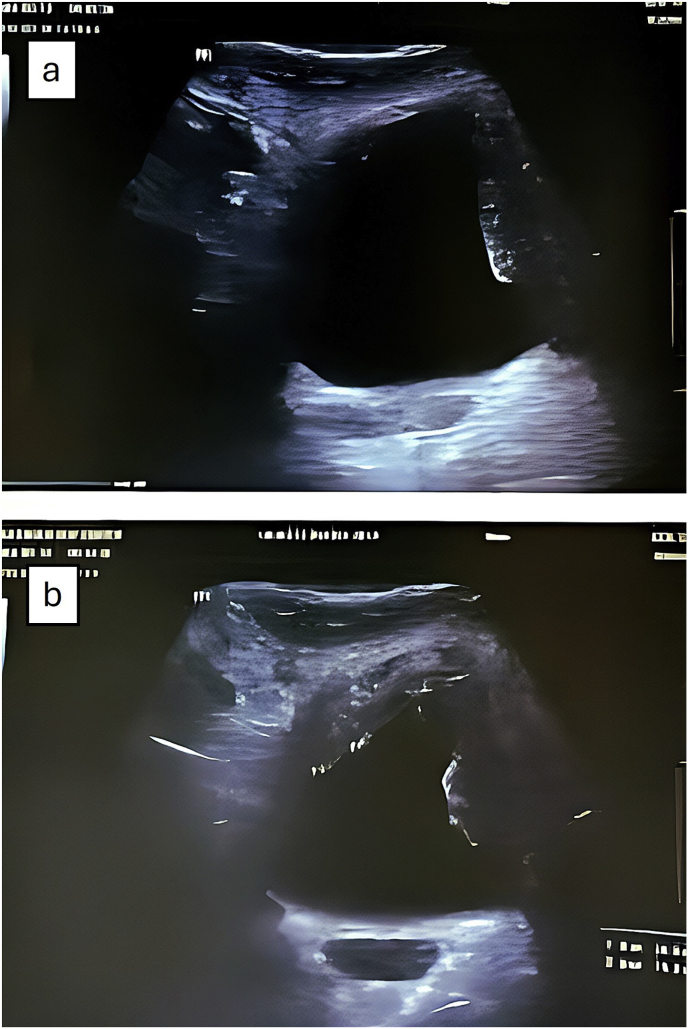
Ultrasonographic evaluation revealed irregular bladder wall thickening with multiple intraluminal projections which is shown in panel A and B in different view. The findings were initially interpreted as suggestive of multifocal bladder tumors.

He presented to our urology clinic with a four-month history of gross hematuria. Ultrasonography of the urinary tract demonstrated multiple large bladder masses with irregular margins (Fig. 1). Following multidisciplinary discussion and clearance from the immunology team, diagnostic cystoscopy with possible biopsy was performed.

**Fig. 2:**
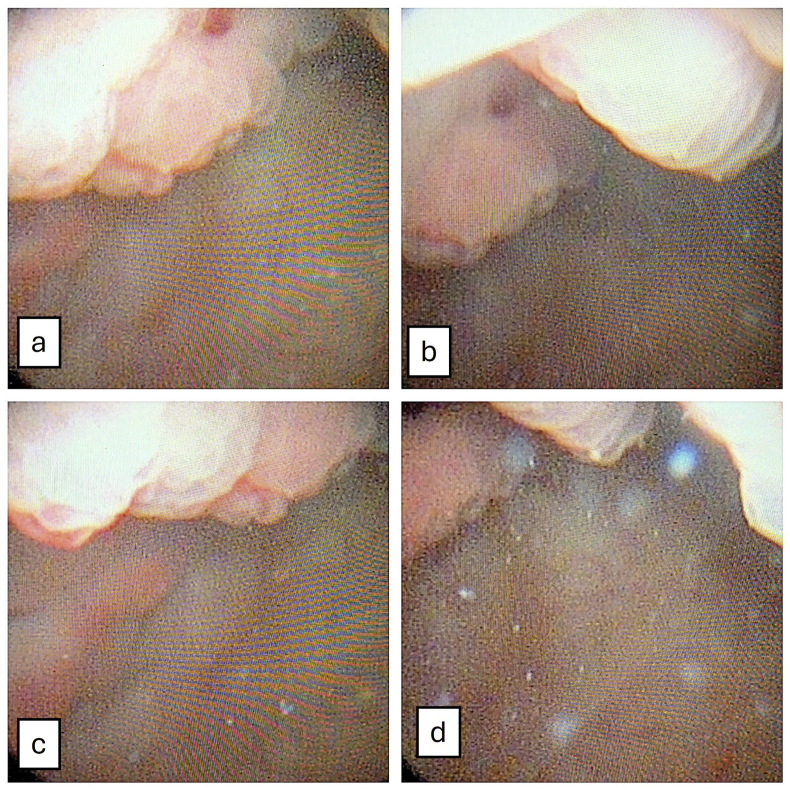
Cystoscopy evaluation demonstrates multiple broad-based, sessile masses diffusely involving the bladder mucosa. The lesions appeared erythematous and friable, resembling urothelial carcinoma. The lesions are shown in panel A to D in different view.

Cystoscopy evaluation (Fig. 2) revealed numerous large, papillary masses diffusely distributed across the bladder mucosa. Because of the extensive involvement and the impracticality of complete resection, representative biopsies were obtained.

Histopathological examination demonstrated papillary urothelial proliferation with mild cytologic atypia, findings compatible with papillary urothelial neoplasm of low malignant potential (PUNLMP). However, due to the limited sample size, definitive distinction between reactive urothelial changes and PUNLMP was not possible, despite multiple reviews by senior pathologists. No lamina propria invasion was identified.

**Fig. 3:**
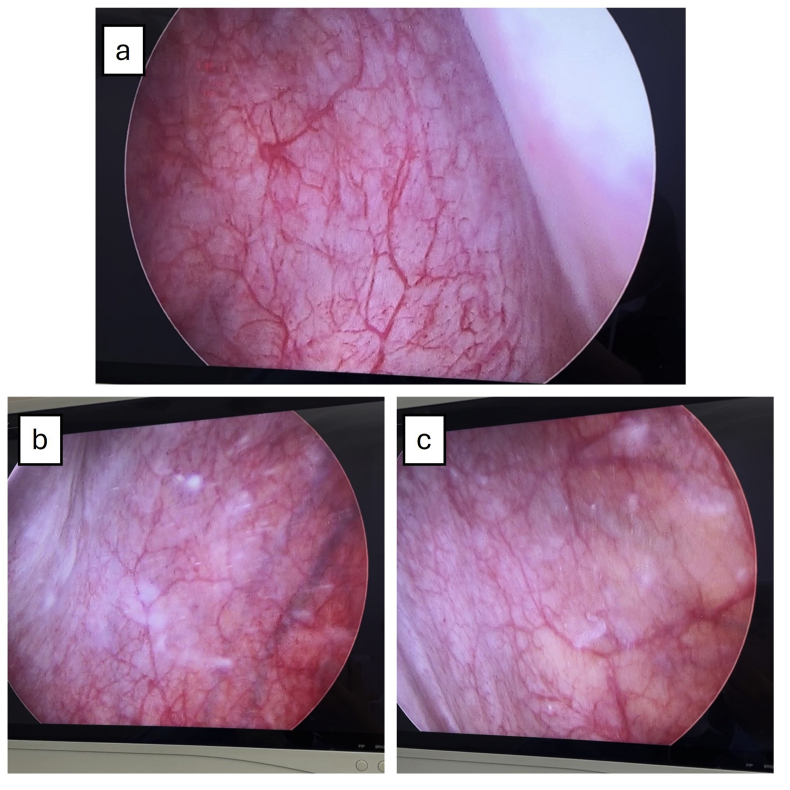
Follow-up cystoscopic view demonstrates normal bladder mucosa with complete resolution of the previously observed lesions. The mucosal surface appears smooth and intact, with no evidence of erythema, edema, or residual mass formation. Normal bladder mucosa is shown in panel A to C in different view.

Given the patient's unusually young age, underlying immunodeficiency, unexpected histopathologic features, and limited biopsy volume, a repeat cystoscopy was planned to obtain deeper and more representative tissue samples. Unexpectedly, at the second cystoscopy one month later, all previously visualized bladder masses had completely resolved. The bladder mucosa appeared entirely normal—smooth, intact, and without any residual lesions or trabeculation (Fig. 3). MRI evaluation further confirmed the complete resolution of the lesions, demonstrating no residual masses within the bladder.

Importantly, during the interval between the two procedures, the patient had not received any new medications beyond his routine cotrimoxazole prophylaxis.

## 3 Discussion

Chronic granulomatous disease (CGD) is a rare inherited immunodeficiency characterized by defective nicotinamide adenine dinucleotide phosphate (NADPH) oxidase function, resulting in impaired microbial killing and a tendency toward granuloma formation.^6^ Although CGD is known to affect multiple organ systems, particularly the lungs, gastrointestinal tract, skin, and lymph nodes, involvement of the genitourinary system remains distinctly uncommon. Urological manifestations generally arise from granulomatous inflammation leading to obstruction of the urinary tract, ureteral strictures, or prostatitis.^3^ Reports of bladder involvement are exceedingly rare, and the appearance of mass-like bladder lesions in patients with CGD has been documented only sporadically in the literature^4^^,^^5^^,^^7^.

In the limited published cases, bladder lesions have typically represented infectious or inflammatory masses rather than true neoplasms. Granulomatous cystitis, fungal infections, and reactive inflammatory proliferations have been described, sometimes mimicking malignancy on imaging and cystoscopy. However, complete regression of bladder masses within a short interval, as observed in our patient, is exceptional. The spontaneous disappearance of the lesions strongly favors a reactive or inflammatory etiology rather than a neoplastic process. This observation aligns with the known propensity of CGD patients to develop exuberant granulomatous or inflammatory tissue responses that may fluctuate or resolve once the underlying stimulus abates.

The initial histopathological findings in our case—papillary urothelial proliferation with mild cytologic atypia^8^ —were concerning for papillary urothelial neoplasm of low malignant potential (PUNLMP). However, the biopsy specimen was obtained via cold-cup technique and was limited in depth and volume. This raises important diagnostic considerations. Small or superficial samples may inadequately capture the architecture and depth of papillary lesions, making it difficult to distinguish between true neoplastic changes and reactive urothelial atypia. Inflammatory conditions associated with immunodeficiency, such as CGD, can further complicate interpretation by producing atypical reactive changes that mimic early neoplasia. The complete normalization of the bladder mucosa on repeat cystoscopy strongly suggests that the initial pathology represented reactive urothelial proliferation rather than true PUNLMP.

Spontaneous regression of bladder masses in Chronic Granulomatous Disease (CGD) occurs because these are inflammatory granulomas, not tumors. Their behavior is inherently volatile—they can swell during active inflammation and then shrink as the body's dysregulated immune response naturally downshifts. Other immune cells may help calm the inflammation, allowing the tissue to be reabsorbed. This shrinkage is a positive sign but does not cure CGD; the risk of future inflammation and mass formation remains.^9^ This case showed complete regression of papillary mass-like lesions without changing in any medication.

Given the rarity of bladder mass-like lesions in CGD, this case highlights the need for clinicians to maintain a broad differential diagnosis when evaluating bladder abnormalities in immunocompromised patients. Awareness of CGD-associated inflammatory mimics is essential to avoid overtreatment or unnecessary interventions. When pathology is inconclusive and clinical suspicion for malignancy is low, short-interval re-evaluation may be a prudent strategy, particularly in young patients with known immunologic disorders.

In summary, this case underscores the importance of considering CGD-related inflammatory processes in the differential diagnosis of bladder masses, recognizing the limitations of small biopsy specimens, and appreciating the potential for spontaneous regression of reactive lesions. It adds to the limited literature describing urological involvement in CGD and emphasizes the value of cautious, individualized management in such rare presentations.

## 4 Conclusion

This case presents an unusual occurrence of transient bladder mass–like lesions in a young patient with chronic granulomatous disease, underscoring how CGD-related inflammatory or reactive urothelial changes can closely resemble malignancy and highlighting the diagnostic limitations of small biopsy samples. CGD-associated inflammatory should be included in the differential diagnosis of bladder masses and may reasonably consider short-interval follow-up when the likelihood of true neoplasia is low in such patients.

## CRediT authorship contribution statement

**Mehdi Dadpour:** Writing – review & editing, Writing – original draft, Supervision, Resources, Project administration, Methodology, Investigation, Conceptualization. **Niloofar Soleimanifard:** Writing – original draft, Methodology, Investigation, Data curation. **Sobhan Alishah:** Writing – original draft, Methodology, Investigation, Data curation. **Miaad Chalaki:** Methodology, Investigation, Data curation.

## Fund

No fund was used in this report.

## Conflict of interests

All authors declare that they have no conflict of interests.
